# Supplementary figures and images for: Publication bias in trials registered in the Australian New Zealand Clinical Trials Registry: Is it a problem? A cross-sectional study
Source: PLoS One. 2023 Jan 5;18(1):e0279926. doi: 10.1371/journal.pone.0279926 (PMC9815633; doi:10.1371/journal.pone.0279926)

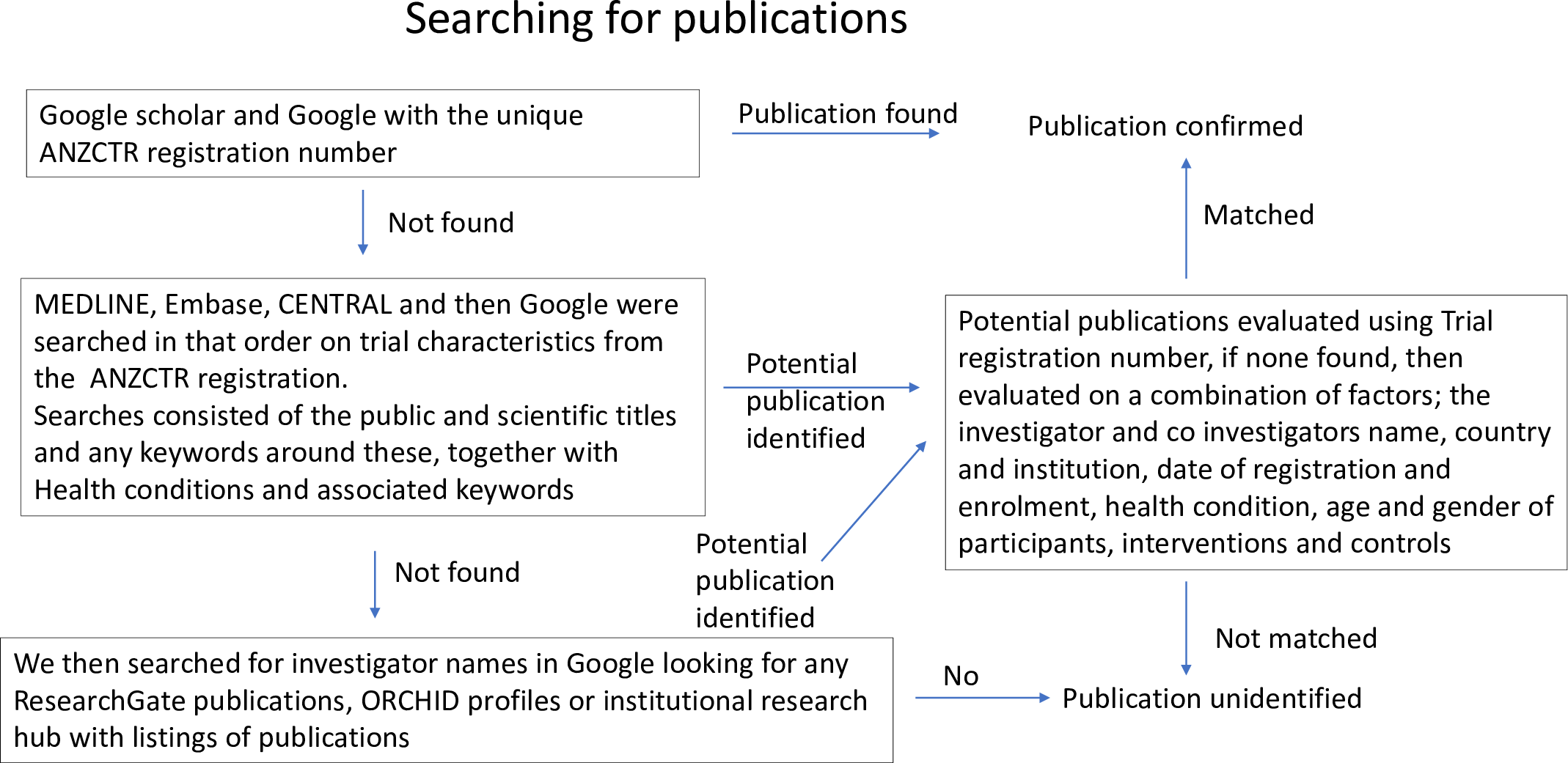

Supplement: S1 Appendix — (TIF) [file pone.0279926.s001.tif]
