# Supplementary material for: Publication bias in trials registered in the Australian New Zealand Clinical Trials Registry: Is it a problem? A cross-sectional study
Source: PLoS One. 2023 Jan 5;18(1):e0279926. doi: 10.1371/journal.pone.0279926 (PMC9815633; doi:10.1371/journal.pone.0279926)
Supplement: Attachment — Submitted filename: Response to Reviewers.docx [file pone.0279926.s003.docx]

Response to Reviewers: revisions manuscript number: PONE-D-22-24255

Thanks for the opportunity to revise this manuscript.

**Reply to the comments from the Journal Editor**

**Comment 1**: “Please ensure that your manuscript meets PLOS ONE's style requirements, including those for file naming

**Response:** I have followed the guidance provided regarding PLOS ONE’s style requirements and made changes as necessary.

**Comment 2:** “In your Data Availability statement, you have not specified where the minimal data set underlying the results described in your manuscript can be found. PLOS defines a study's minimal data set as the underlying data used to reach the conclusions drawn in the manuscript and any additional data required to replicate the reported study findings in their entirety. All PLOS journals require that the minimal data set be made fully available”.

**Response:** I have uploaded our underlying data det as a Supporting Information file S2 (excel spreadsheet) to PLOS ONE

**Comment 3:** “We note that you have stated that you will provide repository information for your data at acceptance”

**Response:** I have now decided to upload the data to PLOS ONE so the repository information is no longer required. I have described these changes in the cover letter.

**Comment 4:** “Please review your reference list to ensure that it is complete and correct”

Response: I have reviewed the reference list and no changes were made.

**Reply to the comments from the reviewers**

**Reviewer one**

**Introduction**

**Comment 1:** “lines 69-70: " We know that the quicker trial results are disseminated, the more clinically relevant these results become (16, 17)" - the authors may wish to clarify what is meant by the term "clinically relevant" when used in this context - are the results more likely to be incorporated in clinical decision making or practice guideline, lead to practice change, or the authors meant something else?”

**Response:** I have reworded this sentence to clearly explain what we meant by “clinically relevant”

**Comment 2: “**lines 102-103: the authors may wish to explain here specifically why these three particular years (2007, 2009 and 2011) were selected for this study.”

**Response:** I have added an explanation for the selected years.

**Data collection**

**Comment 3:** lines 124-125: "We calculated the time to publication in years and months, from the date of anticipated first enrolment, found in the data of the ANZCTR download, to the publication date found in the paper." - what about retrospectively registered trial? Which date was taken as the first point of measurement?

**Response:** Both prospectively and retrospectively registered trials provided a date of first participant enrolment which was taken as the first point of measurement.

**Comment 4: “**lines 142-143: "Any trials that did not describe their RCT design type (parallel, cross over, or factorial) and trials coded as other were considered as one group, 'Other.'" - should they instead be labelled as "unclear" as there was a lack of required information?”

**Response:** I have now changed this category from “Other” to “Unclear”

**Comment 5:** Search Strategy to identify the publication of trials - it will be very helpful for future researchers who wish to do similar work if a generic methodology or algorithm in tracing publication used in this study, if available, be made available either in the main text or in the appendix. This will set a standard for others to follow and possibly update as required.

**Response:** Thank-you for this suggestion. I have now created a pdf of the search algorithm and uploaded it to PLOS ONE as a supplementary file (S1_Appendix).

**Comment 6:** “PLOS authors have the option to publish the peer review history of their article. If published, this will include your full peer review and any attached files.”

**Response:** Yes
