# Supplementary material for: Publication bias in trials registered in the Australian New Zealand Clinical Trials Registry: Is it a problem? A cross-sectional study
Source: PLoS One. 2023 Jan 5;18(1):e0279926. doi: 10.1371/journal.pone.0279926 (PMC9815633; doi:10.1371/journal.pone.0279926)
Supplement: Attachment — Submitted filename: Response to Reviewers.docx [file pone.0279926.s004.docx]

Response to Reviewers: revisions manuscript number: PONE-D-22-24255 14.12.2022

Thanks for the opportunity to revise this manuscript.

**Reply to the comments from the Journal Editor**

I have added 2 more references to the reference list, these are; *The ANZCTR – About Us,* #30 in the reference list and *The ICMJE – Update on Trials registration: Looking Back and moving Ahead June 2007,* #39 in the reference list.

**Reply to the comments from the reviewers**

**Reviewer one**

**Comments 1- 5**: were given a ‘Yes’ by the reviewer.

**Comment 6: “**Thank you for attending to the comments. There remains one point that I would like clarification. In my comment no 2: “lines 102-103: the authors may wish to explain here specifically why these three particular years (2007, 2009 and 2011) were selected for this study.” As far as I can see, the author justified the selection of the years by stating that they wished to allow at least 10 years to assess publication status. That is fine, my point was why picked 2007, 2009 and 2011, i.e. and not including years like 2008 and 2010. I don't seem to have seen any explanation on this’

**Response:** Thank you for pointing this out. I have now provided a full explanation for the choice of these three selected years. It can now be found in the manuscript in the Materials and Methods section under Data source, lines 105 to 115.
